# Circulating vaccine derived polio virus type 1 outbreak, Saadah governorate, Yemen, 2020

**DOI:** 10.1186/s12879-022-07397-0

**Published:** 2022-04-29

**Authors:** Mutahar Ahmed Al-Qassimi, Mohammed Al Amad, Labiba Anam, Khaled Almoayed, Ahmed Al-Dar, Faten Ezzadeen

**Affiliations:** 1 National Polio Surveillance, Yemen Ministry of Public Health and Population, Sana’a, Yemen; 2 Field Epidemiology Training Program, Yemen Ministry of Public Health and Population, Sana’a, Yemen; 3 General Directorate for Diseases Control and Surveillance, Yemen Ministry of Public Health and Population, Sana’a, Yemen

**Keywords:** Circulating vaccine derived polio virus type 1 outbreak, Saadah governorate, Yemen, 2020

## Abstract

**Background:**

Yemen has faced one of the worst humanitarian crises in the world since the start of the war in 2015. In 2020; 30 Vaccine Derived Polio Virus type 1 (VDPV1) isolates were detected in Saadah governorate. The aims are to characterize the outbreak and address the gaps predisposing the emergence and circulation of VDPV1 in Saadah governorate, Yemen.

**Method:**

A retrospective descriptive study of confirmed cases of VDPV1 between January and December 2020 was performed. Surveillance staff collected data from patient cases, contacts, as well as stool specimens that shipped to WHO accredited polio labs. Data of population immunity was also reviewed. The difference in days between the date of sample collection, shipment, and receiving lab result was used to calculate the average of delayed days for lab confirmation.

**Results:**

From January to December 2020, a total of 114 cases of acute flaccid paralysis (AFP) were reported from 87% (13/15) districts, and cVDPV1 was confirmed among 26% (30) AFP cases. 75% (21) were < 5 years, 73% (20) had zero doses of Oral Polio Vaccine (OPV). The first confirmed case (3%) was from Saadah city, with paralysis onset at the end of January 2020 followed by 5 cases (17%) in March from another four districts, 8 cases (27%) in April, and 13 (43%) up to December 2020 were from the same five districts in addition to 3 (10%) form three new districts. The lab confirmation was received after an average of 126 days (71–196) from sample collection. The isolates differ from the Sabin 1 type by 17- 30 VP1 nucleotides (nt) and were linked to VDPV1 with 13 (nt) divergence that isolated in July 2020 from stool specimens collected before one year from contacts of an inadequate AFP case reported from Sahar district.

**Conclusion:**

The new emerging VDPV1 was retrospectively confirmed after one year of sample collection from Sahar district. Delayed lab confirmation, as well as the response and low immunization profile of children against polio, were the main predisposing factors for cVDPV1 outbreak. This outbreak highlights the need to maintain regular biweekly shipments to referral polio labs in the short-term, and the exploration of other options in the longer-term to enable the Yemen National Lab to fully process national samples itself.

**Supplementary Information:**

The online version contains supplementary material available at 10.1186/s12879-022-07397-0.

## Introduction

Oral polio vaccine (OPV), a live vaccine consisting of Sabin attenuated poliovirus strains 1, 2, and 3, is the primary tool for the global eradication of poliovirus. OPV provides both humoral and mucosal immunity, the latter of which makes it effective at interrupting transmission of Polio Viruses (PVs) [1]. PVs are classified as wild-type polioviruses (WPVs), oral polio vaccine viruses (OPVVs), and Vaccine-Derived Polioviruses (VDPVs) which were originally contained in OPV [2, 3].

However, replicating polioviruses have a high frequency of genetic mutation and recombination with other vaccine serotypes and other enteroviruses [4], which may rarely result in vaccine variants reacquiring the ability of the parent wild strains to cause paralytic polio [5, 6]. Sabin oral polioviruses (OPVs), and vaccine-derived polioviruses (VDPVs), which are OPV-related strains that differ from Sabin vaccine strains because they have lost some of their attenuating mutations, so regain their virulence and become able to cause paralytic polio [7]. In settings with low population immunity, Sabin vaccine strains may be transmitted for an extended period among susceptible carriers, increasing the possibility of the emergence and spread of neuro-virulent strains [6, 8, 9].

VDPVs are classified as circulating if there is evidence of community transmission (cVDPV), as immunodeficiency-associated if isolated from a person with an immunodeficiency (iVDPV), or ambiguous if the source is unknown (aVDPV) [2, 10].

Despite the progressive reduction in WPV cases, two countries, Afghanistan and Pakistan still endemic for type 1 WPV (WPV1), and outbreaks of VDPVs have emerged as a major challenge for the final stage of polio eradication efforts. In 2020, 1078 cases of cVDPV2 were confirmed in 24 countries, most of them in Africa [11].

The first documented cVDPV1 outbreak was in 2001, in Hispaniola [12].

Table 1 shows the most recent cVDPV1 outbreaks reported in countries 2015–2020.

**Table 1:** Circulated Vaccine Derived Polio Virus Type 1 Outbreak Reported Countries 2015–2020

Country	Year	Index case paralysis onset	Date of detection or declaration outbreak	Days from date onset to detection	Total cases	Total contacts	Total environmental	Reference
Madagascar	2014–2015	Sep-14	Nov-14	61	10	11		[13]
*Ukraine	2015	Jul-15	Aug-15	58	2	0		[13, 14]
Laos	2015–2016	Sep-15	Oct-15	29	11	25		[15]
Papua New Guinea	2018	Apr-18	May-18	26	26	7	7	[11, 16]
Indonesia	2019	Jan-19	Feb-19	19	1	2		[17, 18] [11, 19]
Myanmar	2019	May-19	Jun-19	32	6	6		
**Philippines	2019	Jul-19	Sep-19	48	2	1	14	
Malaysia	2019–2020	Oct-19	Dec-19	41	4		21	

*Paralysis onset of the second case

**VDPV1 virus isolated first from Environmental samples collected in July 2019

In Yemen, during 2011–2012, nine cases of cVDPV type 2 have been detected by AFP surveillance in six governorates, including; Al Hodydah, Ibb, Hajjah, Amran, Saadah, and Sanaa city [20]. The last detected VDPV was in June 2016; ambiguous type 2 aVDPV2, in a case from Aden governorate [21].

During the period 2011–2016, AFP surveillance met the majority of WHO standard indicators, except for receiving results from reference lab within 28 days from the date of receipt by the lab in 2015; the percentage of specimens with laboratory results was 48% compared to WHO standard timeliness 80% [22].

During 2020 and 2021 both cVDPV1 and cVDPV2 ourbreaks were reported, respectively. The second one started in August 2021 and is still going on [23]. As for the first one, 30 cVDPV1 isolates were detected during 2020 in Saadah governorate.The aims of this paper are to characterize the cVDPV1 outbreak and address the gaps predisposing to the emergence and circulation of VDPV1 in Saadah governorate, Yemen.

A strategic response to a poliovirus event or outbreak according to WHO standard operation procedure [24], provides a timeline and activities to be implemented since day 0, which is the day of receipt of the genetic sequencing laboratory result by WHO. include, Investigating the case or environmental isolate and local context, determining the geographic extent of transmission, risk assessment; virologic risk, and contextual risk. These activities are to be implemented within 72 h.

Surveillance system enhancement, communication, and social mobilization.

The vaccination response in cVDPV1 outbreak includes a rapid response round 0 campaign with bOPV vaccine targeting 200,000–500,000 children below the age of 5 years old to be conducted within 14 days, then within 90 days, two large scale campaigns, SIA1 and SIA2 targeting 1–2 million children below 5 years old in each with no more than one-month separation, a mopping-up campaign after 12 weeks, and an outbreak response assessment.

## Methodology

### Setting and population

Yemen has suffered from political conflicts and war since 2015. Consequently, it has faced one of the worst humanitarian crises in the world. About 80% (24 million) of people need humanitarian assistance, 3.65 million are internally displaced, The health system has collapsed and vaccine-preventable diseases have reemerged.[25, 26] Saadah governorate is one of the most affected governorates by the war. It is located on the northern side of Yemen, about 180 km from the capital, Sana’a. It is administratively divided into 15 districts, with an estimated population of 1,185,169 in 2020. Children < 15 year are 552,741 and under 5 years are 251,011. More than 320,634 people are internally displaced from districts that have active frontlines on borders with Saudi Arabia.

The health system infrastructure is mostly destroyed, routine immunization (RI) coverage is low, and significant numbers of vaccine-preventable disease outbreaks such as diphtheria and measles are reported. [25, 27] Out of 15 districts, 11 districts have accessibility and insecurity issues, (three districts are fully inaccessible, and 8 districts are partially inaccessible).[28, 29].

**Fig. 1:**
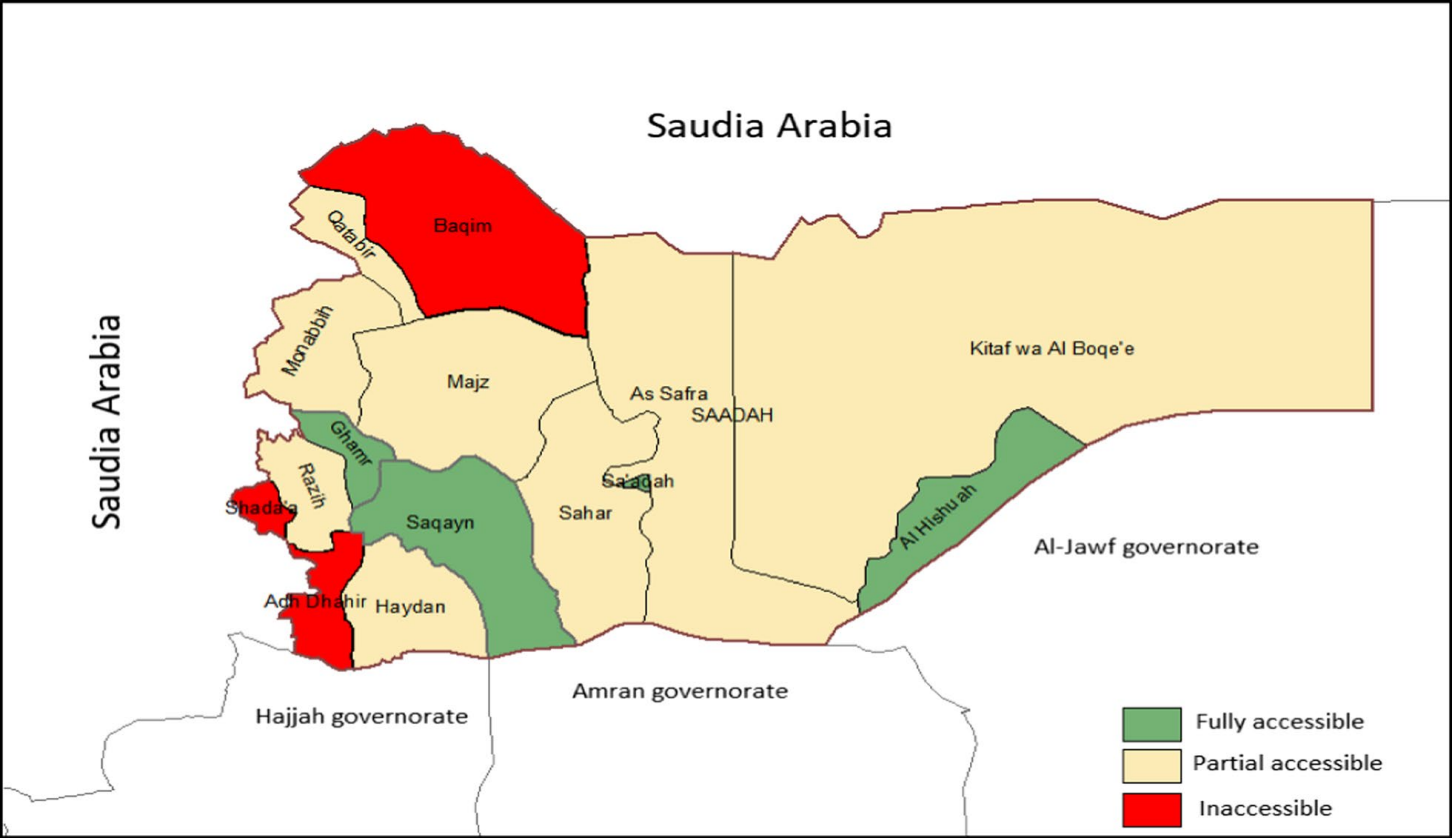
District accessibility of Saadah governorate 2020

Figure 1 shows the district accessibility of Saadah governorate, 2020.

### Case ascertainment and definition

Case patients were identified through the surveillance system for Acute Flaccid Paralysis (AFP), which was established in the country in 1998 with the support of the World Health Organization (WHO). The system activities include; case detection, reporting, investigation, samples collection, active search, and flow-up assessment of AFP cases after 60 days of paralysis onset.

AFP case was defined as a child aged < 15 years with a sudden onset of flaccid paralysis or at any age when poliomyelitis is suspected. Two stool specimens from each AFP case are collected by governorate surveillance officers and sent to the AFP surveillance program in the Ministry of Health. Yemen has no national polio lab, and AFP specimens are sent to WHO referral or accredited polio labs [30]. Recently, stool specimens of all AFP cases were sent to Oman's National Polio Lab (ONPL), where the samples with positive results or (discordant) in intrataypic differentiation tests were sent for genetic sequences to the Centers for Disease Control and Prevention (CDC) in the United States.

The final classification of AFP cases depends on WHO-accredited polio lab results. So polio case is an AFP case when poliovirus is isolated from its sample and the compatible case is a case with inadequate samples with residual paralysis on 60 days follow up and decided by National Polio Expert Committee (NPEC). The non-polio AFP case is either an AFP case with adequate stool samples negative for wild poliovirus and VDPVs or a case with inadequate stool samples discarded by NPEC [31].

The WHO definition for VDPVs was used; an AFP case with genetic sequences from at least one stool sample shows Sabin-related poliovirus divergence (≥ 10% nucleotides (nt) in genomic part VP1) for PV types 1and 3.

The contacts of the AFP case should be close family members or household contacts, and if not possible, then from neighbors or playmates, preferably aged < 5 years [31].

### Epidemiological investigation for VDPV1 cases and close contacts

It was conducted according to WHO Standard Operational Procedures (SOPs) responding to poliovirus event or outbreak [24]. Stool samples were collected from contacts of index VDPV1case, children under 5 years including people who lived in the same household, neighbors, and community contacts.

### Rapid assessment of vaccination coverage

After confirmation of VDPVs cases, a household vaccination survey for OPV coverage was conducted among 30 houses around each VDPV1 case and the OPV status was determined by checking immunization cards. Desk review for data of RI and supplementary immunization activities (SIAs) from 2015 to 2019 was carried out.

### Data collection, management, and analysis

Descriptive epidemiological information on source patients and isolates was collected by surveillance staff. Information on cases, sample collection, and results are kept at national AFP surveillance in the general directorate for diseases control and surveillance.

We conducted a retrospective analysis of confirmed cases from Saadah governorate. Confirmed cases were categorized according to the date of paralysis onset and the date of sample shipments. Differences in days between the dates of sample collection, shipment, and lab result receipt were used to calculate the average, minimum, and maximum delayed days of lab confirmation. Epi-info version 7.2 was used for data entry and analysis.

## Results

**Fig. 2:**
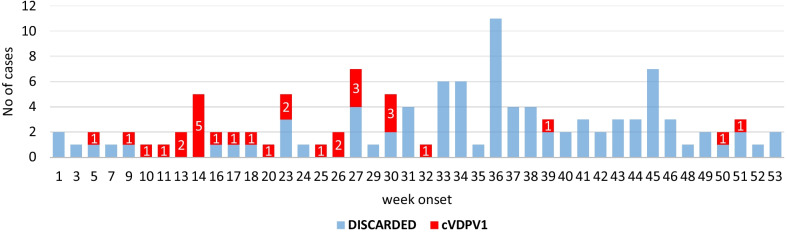
Classification of acute flaccid paralysis cases by week of paralysis onset, Saadah governorate, January–December 2020

From January to December 2020, a total of 114 AFP cases were reported from 13 (87%) out of 15 districts in the Saadah governorate. 26% (30) were confirmed as cVDPV1, and the highest number of confirmed cases were from Sahar district. Figure 2 shows the classification of acute flaccid paralysis cases by district, Saadah governorate, January–December 2020.

### Detection of vaccine-derived polioviruses cases

Out of 30 detected cases of cVDPV, 53% (16) were males, 73% (20) had zero doses of OPV and 75% (21) were less than five years old and the median age was 21 months, ranging from 8 to 144 months. The first detected case (Case 1) was a male aged 72 months (6 years), reported on 1^st^ of February 2020 from Saadah city with paralysis onset on 31 January 2020. A cohort consisted of five cases (Case 2 to Case 6) paralyzed during March 2020 and were from four different districts; two cases (cases 2 and 4) were from As Safra district and three cases were from Sahar, Majz, and Kitaf Wa Albuga districts; one per each. A second cohort consisted of eight cases (Cases 7 –14) paralyzed during April and were almost from the same previously reported districts, three cases were from Sahar, four cases from Kitaf Wa Albuga, and one case was from As Safra district, which raised cVDPV1 isolates in As Safra to three cases (10%). Case 15 was a male, 120 months old, paralyzed on 12–5-2020 and was the first detected case in Saqayn district, which is adjacent to Sahar and Majz districts. The third cohort consisted of six cases (cases 16–21) paralyzed during June 2020; two cases were from Sahar (cases 16 and 20), Kitaf Wa Albuga, case 19 which was the second case from Saadah City district, and case 21 which was the first case (3%) from Munabbih district located on the northwest border of Saadah governorate.

The fourth cohort with five cases (cases 22–26) paralyzed in July 2020, two cases were from Kitaf Wa Albuga (cases 22 and 26) which raised the total confirmed cases in the district to 9 cases (30%), two cases (cases 23 and 24) were from Sahar district and case 25 which was the first case (3%) from Al-Hashwa district, located on the southern border of Saadah governorate. The remaining four cases (Cases 27—30) were paralyzed in August, September, and December, three of whom (one per each month) were from Sahar district that raised the number of cVDPV1 in the district to 11 (37%) while one case ( case 29) was the second (7%) confirmed case from Majz district.

Table 2 shows the circulating vaccine derived poliovirus type1 cases (cVDPV1), Saadah governorate, Yemen 2020.

**Table 2:** Cases of Circulating vaccine derived poliovirus type 1

Case No	paralysis onset		District	Age by month	Sex	*bOPV doses
	Date	Month				
Case 1	31–01-20	Jan	Saadah City	72	Male	1
Case 2	01-03-20	Mar	As Safra	60	Female	0
Case 3	03-03-20		Majz	12	Male	0
Case 4	10-03-20		As Safra	8	Female	0
Case 5	23-03-20		Kitaf Wa Albuga	24	Male	0
Case 6	29-03-20		Sahar	108	Male	0
Case 7	02-04-20	Apr	Sahar	32	Female	0
Case 8	02-04-20		Kitaf Wa Albuga	13	Male	0
Case 9	03-04-20		Sahar	26	Female	2
Case 10	03-04-20		Sahar	17	Female	0
Case 11	03-04-20		Kitaf Wa Albuga	20	Female	0
Case 12	18-04-20		Kitaf Wa Albuga	48	Female	2
Case 13	25-04-20		Kitaf Wa Albuga	15	Male	1
Case 14	30-04-20		As Safra	21	Female	1
Case 15	12-05-20	May	Saqayn	120	Male	0
Case 16	05-06-20		Sahar	8	Female	0
Case 17	02-06-20	Jun	Kitaf Wa Albuga	9	Female	0
Case 18	16-06-20		Kitaf Wa Albuga	18	Male	0
Case 19	22-06-20		Saadah City	18	Female	2
Case 20	24-06-20		Sahar	8	Male	0
Case 21	30-06-20		Munabbih	19	Male	2
Case 22	02-07-20	Jul	Kitaf Wa Albuga	18	Female	0
Case 23	03-07-20		Sahar	48	Male	3
Case 24	21-07-20		Sahar	21	Female	0
Case 25	21-07-20		Alhashwa	42	Male	1
Case 26	26-07-20		Kitaf Wa Albuga	12	Male	0
Case 27	06-08-20	Aug	Sahar	144	Female	1
Case 28	25-09-20	Sep	Sahar	84	Male	0
Case 29	11-12-20	Dec	Majz	72	Male	0
Case 30	16-12-20		Sahar	48	Male	0

*bOPV bivaillent oral polio vaccine (1,3)

Male = 16(53%)

0 dose = 20(73%)

### Sample collection, shipments, and lab results

Due to difficulties of samples transportation from the country to the referral labs, a priority of sending and testing was placed for samples of AFP cases. Based on WHO coordination with UN Humanitarian Air Service (UNHAS), and due to limited allowed weight, some samples were transported through UNHAS trips.The stool samples of detected cases were categorized into five groups according to the date of shipment: which was done every three months. The first case was shipped in March, followed by 15 (cases 2–16), 11 (cases 17–27), one (case 28), and two cases (cases 29–30) that were shipped in June, October, December, and February 2021, respectively. The average time difference between the date of sample collection and sample shipments of all 30 cases was 76 days ranged from 16 to 111 days. Therefore, their results were also received after an average of 126 (71 to 196) days from sample collection. The results have yielded in intrataypic differentiation (ITD), discordant Sabin like 1 and 3 (SL1 and SL3). The isolates' genetic sequences revealed an overall 17–30 VP1 nucleotide (nt) divergence from the Sabin1. The case1 result was received in April 2020 and revealed 20-nucleotide differences in the PV1 genomic part compared to SL1 and was not genetically linked to any previously sequenced VDPV1s. The genetic sequences of 15 confirmed cases (cases 2–16) in July 2020 revealed (17–24) nucleotide divergence in the PV1 genomic region compared to SL1, indicating a cVDPV1 outbreak. Furthermore, all cVDPV1 isolates were linked to VDPV1 isolated from two contacts of an inadequate AFP case (Case 0), for whom genetic sequences revealed 13 nucleotide differences. The samples of the two contacts were retrospectively tested and confirmed in July 2020, after the confirmation of the first case (case 1) and 368 days after their sample collection. The two positive contacts VDPV1 were classified their case (case 0) as cVDPV1 case according to WHO guidelines [2].

Table 3 shows the distribution of confirmed cVDPV1 cases by date of shipment and date of receiving result with the average time delay in days.

**Table 3:** Distribution of confirmed cVDPV1 cases by date of shipment and receiving result, Saadah governorate, Yemen 2020

Detected cases and contacts		Sample Shipment				Time delay in days from SC to SSH	lab Result			Time delay in days from SC to RLR
		No. of cases	Date	Month	Dif. days	Average days (Min–Max)	VP1 nucleotide changes vs # Sabin 1	Date	Month	Average days (Min–Max)
Year 2020	Case 1	1	24–03-20	Mar	0	47	20	27–04-20	April	81
	Cases 2–16	15	24–06-20	June	92	72 (16–110)	21 (17–24)	23–07-20	July	112 (71–196)
	cases 17–27	11	10–10-20	Oct	108	88(49–111)	23 (17–27)	24–12-20	Dec	159 (71–186)
	Case 28	1	27–12-20	Dec	78	79	20	21–01-21	Jan	86 (82–90)
	Cases 29–30	2	09–02-21	Feb	44	52 (48–56	28 (26–30)	15–03-21	Mar	90
	Overall					76 (16–111)	22 (17–30)			126( 71–196)
2019	Case 0	1	30–09-19	Sep	0	85	Neg	16–03-20	Mar	253
	Contacts case 0	2	30–01-20	Jan	122	204	13	12–07-20	July	368

*Dif.* difference, *SC* sample collection, *SSH* sample shipment, *RLR* Received lab result

### The result of the epidemiological investigation

The filed investigation for the first detected case (Case 1) showed that the child had been displaced with his family from Hydan district to Saadah city for 3 years, According to the classification and reporting of vaccine-derived polioviruses (VDPV)-GPEI guidelines, the case was assessed by medical and pediatrician doctors, a detailed history and physical examination were conducted, and the result indicated no evidence of primary immunodeficiency disease.

Primary ITD results for stool samples collected from 26 households and community contacts of the index VDPV1 case revealed non-polio enteroviruses (NPEV) among 13 contacts and negative for the rest (Additional file 1).

## Result of immunization coverage

**Fig. 3:**
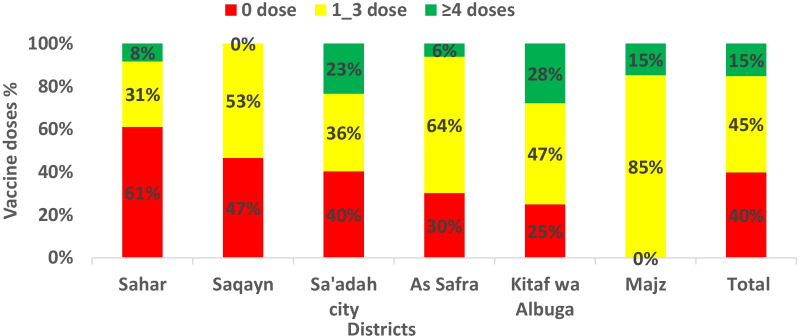
Doses of oral polio vaccine among contacts of cVDPV1 cases by districts,Saadah governorate 2020 (n = 369)

396 children below the age of 5 years from contacts of confirmed cases in six districts were surveyed for OPV immunization coverage; by routine and campaigns, e.g.Supplementary immunization activities (SIAs). The coverage was poor as 51% of surveyed children were unvaccinated by routine vaccination and 66% of children were not vaccinated in the last 4 rounds campaigns, 40% (158) of surveyed children had zero doses (un-immunized), 45% (179) had 1–3 doses (under-immunized) and only 15% (69) had ≥ 4 doses (immunized). A higher percentage of zero doses was 61% and 47% in Sahar and Saqayn districts, respectively (Fig. 3).

The RI coverage by third dose (OPV) was < 50% in three years; 2015, 2017, and 2018, and 61%,74% in two years; 2016 and 2019 respectively. From the nine rounds of SIAs that have been implemented in the country during 2015 -2019, only six SIAs were implemented in the six districts.

**Fig. 4:**
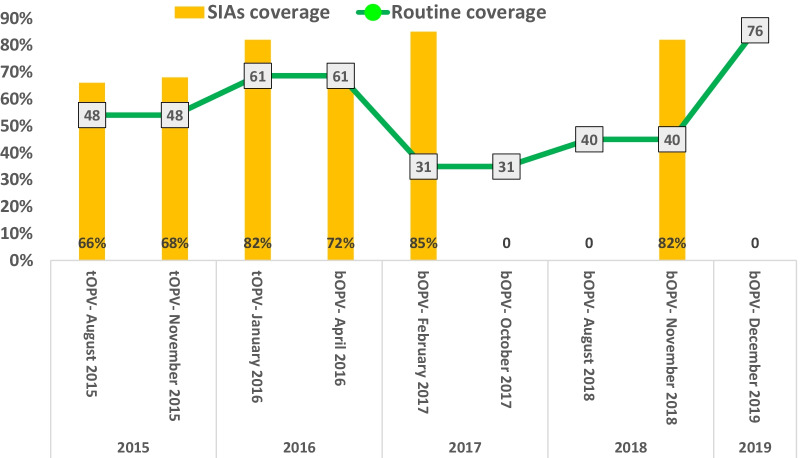
Polio Immunization coverage for Routine and Supplementary activities in Saadah governorate 2015- 2019. *SIAs* Supplementary activities, *tOPV* trivalent oral polio vaccine, *bOPV* bivalent oral polio vaccine

Figure 4 shows polio immunization coverage for routine and supplementary activities in Saadah governorate, (2015- 2019).

## Discussion

This study provides information on the cVDPV1 outbreak in one of the Eastern Mediterranean conflict countries, such as Yemen. This country has suffered from war since 2015. Consequently, the health system has collapsed, vaccine-preventable diseases have reemerged, and polio eradication activities in both components: surveillance system and immunization have been interrupted.

This paper describes 30 cVDPV1 that were isolated from 114 AFP cases that have been reported during 2020 from all districts of Saadah governorate except two districts. Shada district is inaccessible, and Razih district is partially accessible. The two districts are on the borders of Saudi Arabia and have active frontlines, and the majority of their populations were displaced to other districts that are located in the center of the governorate [32–34]. This is also the explanation for the lower number of reported AFP cases from the other such districts and the higher number of reported AFP cases from districts in the center.

The majority of lab-confirmed cVDPV1 were zero doses, and the rest were under-3 doses. This finding is compatible with other recorded cVDPV1 outbreaks, such as the China cVDPV1 outbreak in 2002 [35]. Almost three-quarters of cases were under the age group of 5 years, approximately the same as the Papua New Gina cVDPV1 outbreak in 2018, where 73% of positive cases were under five [16].

However, for cVDPV to occur, it must circulate in an unvaccinated or under-immunized population for a period of at least 12 months [6, 19]. In contrast, our findings showed frequent detection of cases as a single case or cohort of cases: after a month of the first case that appeared in Saadah city at the end of January 2020, 5 cases appeared in four new districts, followed by eight cases in the next month, almost within the same districts. This result indicated that the detected cases in Saadah city district might not be the first cases in this outbreak. Furthermore, our study showed a genetic sequence between the confirmed cases and another case that was detected in 2019 in Sahar district. It has indicated more than one year of circulation and is considered as a new emerging VDPV1 [6, 36].

Our findings showed that during May–July, 3 cases have been detected in three new districts (one per month), while the rest (13 cases) and up to December 2020 were frequented by the old five districts. This might be due to a delay in response in five districts where the virus is spreading through person-to-person transmission within the same district and to the adjacent districts where a new single case has appeared at the beginning.

According to the findings, more than a third of the cases were from Sahar, a third from Kitaf Wa Albuga, and a variable percentage from other districts. This might be due to high population density; the majority of displaced people are hosted in the central districts, and the high population movement between adjacent districts like Sahar, Kitaf Wa Albuga, and As Safrah [29, 33].

Similar to the results of two studies that evaluated the performance of AFP surveillance in Yemen from 2010 to 2017 [22, 37]. Our results showed a considerable delay in lab confirmation after sample collection for the majority of cases. The finding has shown a long period between sample collection and shipment as well as receiving results, with an average of > 4 months for detected cases in 2020 and one year for cases in 2019. The challenges in the shipment of AFP stool samples, the unavailability of a national polio lab in Yemen, the pan, and the closure of Sana'a airport are the explanations for this result.

The finding of this study has presented that; the first detected case in 2020 was after 7 months with (17–24) nucleotide divergence from isolated VDPV1 in July 2019 13 (nt). The nucleotide divergence since the first detection didn’t show orphan viruses, which indicated no gaps in surveillance [31].

The lab results of 26 households contact samples of the index VDPV1case were negative. The reason for this result could be due to the delayed lab result of the first confirmed case (case 1), as well as the that has been performed 4 months apart from paralysis and since poliovirus can shed from an infected person no more than 2 months [31] On the other hand, the result of (case 30) had 30 (nt) divergence that indicated prolonged circulation, reflecting a delayed and inadequate response.

The result showed very poor immunization coverage in the five affected districts for both: routine OPV and SIAs. The chronic under-vaccination situation created favorable conditions for VDPV1 to emerge and circulate, leading to the outbreak [38–41].

There are some limitations to this study. It did not address the response to the epidemic as it was late and limited due to the security situation and the ongoing war, Nevertheless, the current study provides information about the cVDPV1 outbreak in Saadah governorate, Yemen. It could help public health stakeholders to initiate strategic plans for early lab confirmation as well as early response and control of emerging VDPVs in conflict areas.

## Conclusion

The new emerging VDPV1 was confirmed after one year of circulation. Sahar district was the source of cVDPV1 in Saadah governorate and the distribution pattern from Sahar districts to neighboring districts is obvious to extend of the virus to other districts could take place, even to other governorates, especially with poor immunization coverage. Delayed lab confirmation, low immunization profile of children against polio, and delayed response are the main gaps for spreading the virus.

## Recommendation

Regular biweekly shipments to referral polio labs should be made, and considering other options, such as providing Yemen National Lab with the polio direct detection method, poliovirus RNA extraction using real-time reverse transcription (RT)-PCR, which the WHO has recently used in some countries [42, 43], will significantly reduce the number of samples sent to referral labs. High coverage levels of RI should be maintained in children until polio is globally eradicated and be enhanced by high-quality SIAs to cover any immunization gaps, especially in high-risk areas to prevent the emergence or importation and circulation of cVDPVs. High-risk areas, such as conflict zones and disasters, should be targeted with special plans and early interventions within the global plans to eradicate polio. Advocacy, community leadership mobilization, and community involvement in health practices are the key factors for their success.

## Supplementary Information

**Additional file 1: Table S1.** Delayed time for sample shipment and lab result received for confirmed cVDPV1 cases

## Data Availability

All relevant data are presented in this paper, and more information can be provided upon reasonable request from the corresponding author.

## Abbreviations

AFP: Acute flaccid paralysis
aVDPV: Ambiguous vaccine-derived poliovirus
bOPV: Bivalent oral polio vaccine
CDC: Centers for Disease Control and Prevention
cVDPV: Circulating vaccine-derived poliovirus
ITD: Intrataypic differentiation
iVDPV: Immunodeficiency-related vaccine-derived poliovirus
GPEI: Global Polio Eradication Initiative
NPEC: National Polio Expert Committee
Nt: Nucleotides
ONPL: Oman's National Polio Lab
OPV: Oral polio vaccine
RI: Routine immunization
SIAs: Supplementary immunization activities
SL1: Sabin like 1
SL3: Sabin like 3
SOPs: Standard Operational Procedures
tOPV: Trivalent oral polio vaccine
VDPV1,2: Vaccine-derived poliovirus type 1, 2
WHO: World Health Organization
