# Supplementary material for: Circulating vaccine derived polio virus type 1 outbreak, Saadah governorate, Yemen, 2020
Source: BMC Infect Dis. 2022 Apr 29;22:414. doi: 10.1186/s12879-022-07397-0 (PMC9052627; doi:10.1186/s12879-022-07397-0)
Supplement: Supplementary file 1 — Additional file 1: Table S1. Delayed time for sample shipment and lab result received for confirmed cVDPV1 cases [file 12879_2022_7397_MOESM1_ESM.docx]

Additional file 1: Table S1. Delayed time for sample shipment and lab result received for confirmed cVDPV1 cases

| **Case No.** | **Date of sample collection** | **Date of Sample shipment** | | **Difference in days *** | **Date pf lab result received** | | | **Difference in days **** | **Total delayed days ***** |
| --- | --- | --- | --- | --- | --- | --- | --- | --- | --- |
|  |  |  |  |  | **Result** | **Date** | **Month** |  |  |
|  |  | **Date** | **Month** |  | **VP1 nucleotide changes vs #Sabin 1** |  |  |  |  |
| Case 1 | 06-02-20 | 24-03-20 | Mar | 47 | 20 | 27-04-20 | April | 34 | 81 |
| Case 2 | 06-03-20 | 24-06-20 | June | 110 | 19 | 23-07-20 | July | 29 | 139 |
| Case 3 | 11-03-20 | 24-06-20 |  | 105 | 20 | 29-07-20 |  | 35 | 140 |
| Case 4 | 16-03-20 | 24-06-20 |  | 100 | 22 | 23-07-20 |  | 29 | 129 |
| Case 5 | 29-03-20 | 24-06-20 |  | 87 | 24 | 23-07-20 |  | 29 | 116 |
| Case 6 | 02-04-20 | 24-06-20 |  | 83 | 20 | 23-07-20 |  | 29 | 112 |
| Case 7 | 06-04-20 | 24-06-20 |  | 79 | 21 | 23-07-20 |  | 29 | 108 |
| Case 8 | 08-04-20 | 24-06-20 |  | 77 | 21 | 23-07-20 |  | 29 | 106 |
| Case 9 | 05-04-20 | 24-06-20 |  | 80 | 21 | 23-07-20 |  | 29 | 109 |
| Case 10 | 11-04-20 | 24-06-20 |  | 74 | 21 | 23-07-20 |  | 29 | 103 |
| Case 11 | 11-04-20 | 24-06-20 |  | 74 | 23 | 23-07-20 |  | 29 | 103 |
| Case 12 | 01-05-20 | 24-06-20 |  | 54 | 23 | 23-07-20 |  | 29 | 83 |
| Case 13 | 29-04-20 | 24-06-20 |  | 56 | 22 | 23-07-20 |  | 29 | 85 |
| Case 14 | 10-05-20 | 24-06-20 |  | 45 | 21 | 23-07-20 |  | 29 | 74 |
| Case 15 | 19-05-20 | 24-06-20 |  | 36 | 23 | 29-07-20 |  | 35 | 71 |
| Case 16 | 08-06-20 | 24-06-20 |  | 16 | 17 | 21-12-20 | Dec | 180 | 196 |
| Average of delayed days (min-max)) | | |  | 72 (16-110) |  |  |  | 40 (29-180) | 112 (71-196) |
| Case 17 | 29-06-20 | 10-10-20 | Oct | 103 | 23 | 24-12-20 | Dec | 75 | 178 |
| Case 18 | 21-06-20 | 10-10-20 |  | 111 | 24 | 24-12-20 |  | 75 | 186 |
| Case 19 | 29-06-20 | 10-10-20 |  | 103 | 22 | 24-12-20 |  | 75 | 178 |
| Case 20 | 28-06-20 | 10-10-20 |  | 104 | 23 | 24-12-20 |  | 75 | 179 |
| Case 21 | 07-07-20 | 10-10-20 |  | 95 | 21 | 24-12-20 |  | 75 | 170 |
| Case 22 | 09-07-20 | 10-10-20 |  | 93 | 25 | 24-12-20 |  | 75 | 168 |
| Case 23 | 13-07-20 | 10-10-20 |  | 89 | 17 | 24-12-20 |  | 75 | 164 |
| Case 24 | 26-07-20 | 10-10-20 |  | 76 | 25 | 24-12-20 |  | 75 | 151 |
| Case 25 | 27-07-20 | 10-10-20 |  | 75 | 25 | 24-12-20 |  | 75 | 150 |
| Case 26 | 30-07-20 | 10-10-20 |  | 72 | 27 | 27-12-20 |  | 78 | 150 |
| Case 27 | 18-08-20 | 10-10-20 |  | 49 | 26 | 28-10-20 | Oct | 22 | 71 |
| Average of delayed days (min-max)) | | |  | 88(49-111) |  |  |  | 70 (22-78) | 159 (71-186) |
| Case 28 | 09-10-20 | 27-12-20 | Dec. | 79 | 20 | 21-01-21 | Jan-21 | 25 | 104 |
| Case 29 | 15-12-20 | 09-02-21 | Feb-21 | 56 | 26 | 15-03-21 | Mar21 | 34 | 90 |
| Case 30 | 23-12-20 | 09-02-21 |  | 48 | 30 | 15-03-21 |  | 34 | 82 |
| Average of delayed days (min-max) | | | | 52 (48-56 |  |  |  | 34 | 86 |
| Overall Average of delayed days (min-max) | | | | 76 (16-111) |  |  |  | 50 (22-180) | 126( 71-196 |
|  |  |  |  |  |  |  |  |  |  |
| Case 0 | 07-07-19 | 30-09-19 | Sep-19 | 85 |  | 16-03-20 | Mar. | 168 | 253 |
| Contact01 | 10-07-19 | 30-01-20 | Jan-20 | 204 | 13 | 12-07-20 | July | 164 | 368 |
| Contact02 | 10-07-19 | 30-01-20 | Jan-20 | 204 | 13 | 12-07-20 | July | 164 | 368 |
| Average of delayed days (min-max)) | | |  | 164(85-204 |  |  |  | 165(164,168 | 330 (253-369) |

*diffrence in days between date of smaple collection and date of shipmment

**diffrence in days between date of sample shipments and date of lab result received

***difference in days between date of sample collection and date of lab result received
